# The Burden of Disease due to COVID-19 (BoCO-19): A study protocol for a secondary analysis of surveillance data in Southern and Eastern Europe, and Central Asia

**DOI:** 10.1371/journal.pone.0292041

**Published:** 2023-10-13

**Authors:** Caoimhe Cawley, Jonila Gabrani, Aleksandar Stevanović, Rakhat Aidaraliev, Mehtap Çakmak Barsbay, Seila Cilovic Lagarija, Kairat Davletov, Tolkun Djamangulova, Natalya Glushkova, Matthias an der Heiden, Pranvera Kaçaniku-Gunga, Maia Kereselidze, Besfort Kryeziu, Khorolsuren Lkhagvasuren, Samir Mehdiyev, Dariia Oharova, Diloram Sadikkhodjayeva, Milena Santric Milicevic, Milica Stanisic, Stela Stojisavljevic, Gulcan Tecirli, Natasa Terzic, Annelene Wengler, Alexander Rommel, for the BoCO-19 Study Group

**Affiliations:** 1 Department2: Epidemiology and Health Monitoring, Robert Koch Institute, Berlin, Germany; 2 National Association of Public Health, Tirana, Albania; 3 Institute of Social Medicine, Faculty of Medicine, University of Belgrade, Belgrade, Serbia; 4 Public Association “Healthy Future”, Bishkek, Kyrgyzstan; 5 Faculty of Economics and Administrative Sciences, Department of Health Management, Ankara Hacı Bayram Veli University, Ankara, Türkiye; 6 Institute of Public Health of the Federation of Bosnia and Herzegovina, Sarajevo, Bosnia and Herzegovina; 7 Asfendiyarov Kazakh National Medical University, Almaty, Kazakhstan; 8 Faculty of Medicine, Al-Farabi Kazakhs National University, Almaty, Kazakhstan; 9 Department 3: Infectious Disease Epidemiology, Robert Koch Institute, Berlin, Germany; 10 National Institute of Public Health of Kosovo, Pristina, Kosovo; 11 National Center for Disease Control & Public Health, Tbilisi, Georgia; 12 Mongolian National University of Medical Sciences, School of Public Health, Ulaanbaatar, Mongolia; 13 Public health and reforms center, Ministry of Health, Baku, Azerbaijan; 14 Public Health Center of the Ministry of Health of Ukraine, Kyiv, Ukraine; 15 Tashkent Institute of Postgraduate Medical Education, Tashkent, Uzbekistan; 16 Institute of Public Health of Montenegro, Podgorica, Montenegro; 17 Public Health Institute of the Republic of Srpska, Banja Luka, Bosnia and Herzegovina; 18 Ministry of Health, Ankara, Türkiye; Tehran University of Medical Sciences, ISLAMIC REPUBLIC OF IRAN

## Abstract

**Introduction:**

The COVID-19 pandemic has had an extensive impact on public health worldwide. However, in many countries burden of disease indicators for COVID-19 have not yet been calculated or used for monitoring. The present study protocol describes an approach developed in the project “The Burden of Disease due to COVID-19. Towards a harmonization of population health metrics for the surveillance of dynamic outbreaks” (BoCO-19). The process of data collection and aggregation across 14 different countries and sub-national regions in Southern and Eastern Europe and Central Asia is described, as well as the methodological approaches used.

**Materials and methods:**

The study implemented in BoCO-19 is a secondary data analysis, using information from national surveillance systems as part of mandatory reporting on notifiable diseases. A customized data collection template is used to gather aggregated data on population size as well as COVID-19 cases and deaths. Years of life lost (YLL), as one component of the number of Disability Adjusted Life Years (DALY), are calculated as described in a recently proposed COVID-19 disease model (the ‘Burden-EU’ model) for the calculation of DALY. All-cause mortality data are collected for excess mortality sensitivity analyses. For the calculation of Years lived with disability (YLD), the Burden-EU model is adapted based on recent evidence. Because Covid-19 cases vary in terms of disease severity, the possibility and suitability of applying a uniform severity distribution of cases across all countries and sub-national regions will be explored. An approach recently developed for the Global Burden of Disease Study, that considers post-acute consequences of COVID-19, is likely to be adopted. Findings will be compared to explore the quality and usability of the existing data, to identify trends across age-groups and sexes and to formulate recommendations concerning potential improvements in data availability and quality.

**Discussion:**

BoCO-19 serves as a collaborative platform in order to build international capacity for the calculation of burden of disease indicators, and to support national experts in the analysis and interpretation of country-specific data, including their strengths and weaknesses. Challenges include inherent differences in data collection and reporting systems between countries, as well as assumptions that have to be made during the calculation process.

## Introduction

The COVID-19 pandemic has had an extensive impact on public health worldwide. Globally, as of 16 August 2023, there have been 769.806.130 confirmed cases of COVID-19, including 6.955.497 deaths, reported to WHO [1]. In addition to the temporary overloading of hospitals due to high hospitalisation rates, a persistent problem is that approximately 10–20% of infected individuals suffer symptoms of Long COVID, a proportion that has already been estimated to be around 17 million affected individuals worldwide by the end of 2022 [2].

Various indicators can be used to attempt to quantify and compare the health impacts of COVID-19 between countries. Burden of disease metrics have proven to be especially valuable to comprehensively estimate the impact of various conditions on population health, and are calculated in the Global Burden of Disease study (GBD) [3–5], as well as in burden of disease studies at the national Level [6–12]. Burden of disease indicators include YLL, which summarise the number of Years of Life Lost due to death, and YLD, which summarise the number of Years Lived with Disability (or time lost due to ill-health). YLL and YLD can be added together to quantify the overall life years lost due to both mortality and morbidity, in the combined indicator DALY (Disability Adjusted Life Years) [13].

The project “The Burden of Disease due to COVID-19: towards a harmonization of population health metrics for the surveillance of dynamic outbreaks” (BoCO-19) is a collaborative platform, bringing together international public health experts with the aim of scientific exchange, capacity building and joint research implementation. Within BoCO-19, a study is implemented in order to estimate the burden of COVID-19 across 14 countries and sub-national regions in Southern and Eastern Europe (including Türkiye and Ukraine) and Central Asia (including Georgia and Azerbaijan), based on a uniform, comparable and harmonized methodology [14, 15]. Many of these countries have experienced a considerable impact from the COVID-19 pandemic [16, 17], though in most of them burden of disease indicators for COVID-19 have not yet been calculated.

In comparison to standard epidemiological indicators such as disease incidence or prevalence, or rates of hospitalization or death, a strength of burden of disease indicators is that they measure the combined impact of both morbidity and mortality [18]. Burden of disease indicators also enable a comparison of the impact of different conditions on a unified scale (that is, DALY or the number of life-years lost). Not only can the burden of disease be compared between diseases, but also between countries, sub-national regions, or socio-economic strata. Thus, population groups which bear the greatest burden can be identified, and such information can be fed into health information systems in order to inform public health decisions and policy making.

To calculate the burden for a certain condition, a specific model shall be developed, based on which calculations can be made. These disease models entail key methodological decisions, including for example the choice of remaining life expectancy (for the calculation of YLL), and the definition of disease severities and duration of the illness (for the calculation of YLD) [13, 19]. Until now (August 2023), within the GBD study a full disease model for COVID-19 has not been published, nor has the new condition been included into its latest 2019 edition. To nevertheless take advantage of the benefits of burden of disease metrics for pandemic monitoring, different approaches for calculating the burden of COVID-19 have been applied by research teams at the national level [20–27]. While many of these studies refer to a disease model that was recently proposed by the European Burden of Disease Network [28, 29] (the ‘Burden-EU’ model), in practice certain variations in the definitions of severity grades and other model parameters can be found.

Within the present study, the existing Burden-EU COVID-19 model will be further developed in light of new knowledge relating to the calculation of the burden of COVID-19. The analyses will be based on nationally available data, which have not already been routinely reported in the necessary differentiation to international information systems such as those of the World Health Organization (WHO). It is worth pointing out that this study protocol does not describe a primary data collection, but rather the ongoing process of collecting and aggregating existing information, as well as the methodological developments, and the analytical approaches and challenges of the secondary data analyses planned herewith in order to harmonize and implement the burden of COVID-19 calculations across all partner countries.

## Materials and methods

### Aim and objectives

The aim of the study that is implemented in BoCO-19 is to develop and apply a harmonized methodology to calculate the burden of disease due to COVID-19 (as expressed by YLL, YLD, and DALY), in cooperation with 14 different partner institutions from Southern and Eastern Europe and Central Asia. This methodology is not limited in its informative value to the countries and regions involved, but is intended to be generalizable and universally applicable, subject to possible new findings on the COVID-19 pandemic that may necessitate future adaptations of the underlying assumptions.

The specific objectives of the burden of disease study implemented in BoCO-19 are:

- To adapt and refine existing approaches for calculating the burden of COVID-19, such that the methodology can be applied in a cross-country analysis.
- To review, adjust and collate existing time differentiated COVID-19 morbidity and mortality data in each of the partner countries, including consideration of their strengths and weaknesses.
- To implement the developed methodologies and assess the burden of COVID-19 in all partner countries, using the indicators YLL, YLD and DALY, with disaggregation by sex, age-group and over time.

### Study design and setting

**Fig 1:**
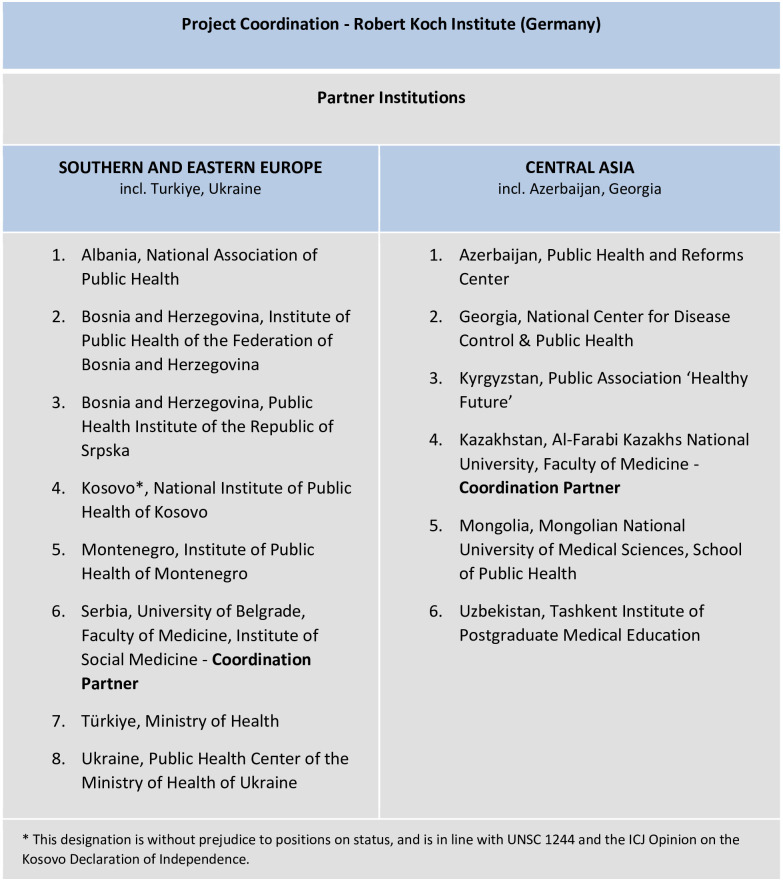
BoCO-19 partner institutions.

The BoCO-19 project is a trilateral cooperation between i) the Robert Koch Institute (RKI), ii) the EU COST Action CA18218 (the European Burden of Disease Network—a collaborative platform which strengthens capacity in burden of disease assessment across Europe) and iii) 14 different partner institutions from the target regions (Fig 1). BoCO-19 was funded for a two-year period from May 2021 to April 2023 under a 2020 ‘Corona Global’ scheme which facilitates international cooperation and coordinated responses to the COVID-19 pandemic, as part of the German Federal Ministry of Health’s Global Health Protection Programme. After the funding period, the final work is currently (August 2023) still being continued. The partners are mainly governmental organizations that fulfil the role of National Public Health Institutes. In some countries, well-known university institutes or non-governmental organizations with good access to the relevant data holders could be won as partners.

The project is coordinated by the RKI, with regional coordination support from the University of Belgrade, Serbia and Al-Farabi Kazakhs National University in Almaty, Kazakhstan. The coordinators are collectively responsible for planning the study activities and the timeline and for supporting activities such as delivering capacity building and training workshops on burden of disease methodology, in collaboration with experts from the European Burden of Disease Network. Project workshops were focused either on conveying basic theoretical knowledge, or designed as practical application workshops (so-called data labs, -see more in detail below). In-country partners take part in working groups and meetings, and are responsible for collecting the required input data, calculating the burden of disease indicators, and contributing to the write-up of study results and scientific publications.

BoCO-19 is also supported by regional and if needed by country offices of the World Health Organization (WHO), primarily in terms of supporting access to data, and communication with national authorities in individual countries, where required. Scientific exchange between stakeholders within the project is facilitated via online meetings, collaborative in-person workshops, as well as dedicated working groups addressing specific topics.

### Status and timeline of the study

**Fig 2:**
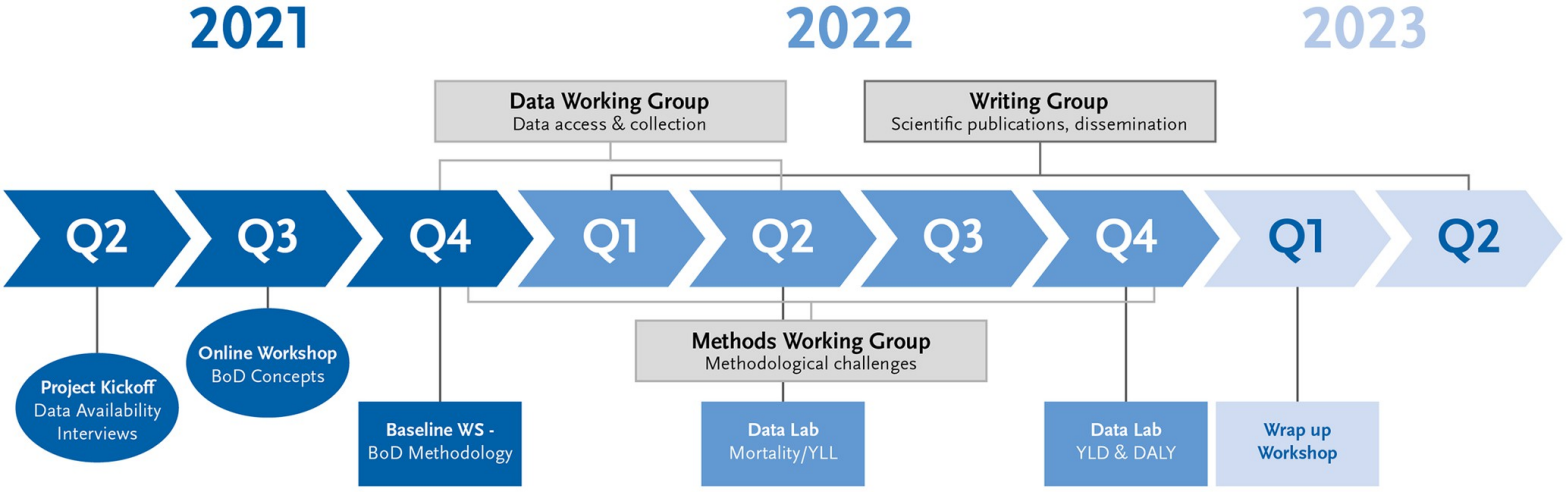
Study timeline and supporting activities within the BoCO-19 project. BoD = Burden of Disease.

As shown in the study timeline and supporting activities, a data availability questionnaire was filled out by all partner institutions at the beginning of the study (Fig 2), followed by individual partner meetings to discuss potential data sources within each country. Other topics were also discussed, such as the time delay with which COVID-19 data become available, the frequency of reporting (i.e. on a daily, weekly, monthly basis) and the sub-national level at which data can be disaggregated. Subsequently, during an online workshop in September 2021 and an in-person workshop held in Belgrade, Serbia in November 2021, burden of disease concepts and methodology were presented and discussed, including specific challenges relating to the development of a harmonized methodology to calculate the burden of COVID-19. The Belgrade workshop resulted in the formation of three Working Groups: a Data Working Group, a Methods Working Group and a Writing Group. These groups work in parallel in order to guide and facilitate the implementation of the study in BoCO-19.

The Data Working Group discusses and proposes solutions for issues relating to data access and collection. As part of the work, a data collection template was created which allows for the collection of data on COVID-19 cases and deaths until March 2021 by all partners in a uniform way. These data were used at two ‘Data Labs’ in 2022, during which a refined methodology was applied mainly to the 2020 data in order to put the underlying concepts into practice and to calculate YLL, YLD and DALY. A second data request to the participating countries is ongoing until September 2023 to compile information for the whole year 2021. The Methods Working Group addresses specific challenges relating to the choice of COVID-19 disease model and the corresponding input parameters, proposing potential approaches and reaching solutions in a collaborative manner. The Writing Group is responsible for considering how study activities and results can best be communicated, and leads the work on scientific publications and other knowledge translation activities.

In spring 2023 a wrap-up workshop was conducted, during which cross-country comparisons of results and their interpretation were discussed, as well as country-specific challenges in data access and data quality, for which recommendations were formulated. Approaches for further dissemination activities were also explored, such as mechanisms to foster the integration of results into national health information systems and communication to policy makers and other stakeholders.

### Data sources and data collection

#### Data sources

National surveillance data on COVID-19 cases are collected in all partner countries by governmental agencies as part of a mandatory reporting on notifiable diseases (Table 1). Data on COVID-19 deaths are available either from the aforementioned surveillance systems, or from national civil registration and vital statistics systems (Table 2). For both COVID-19 cases and deaths, there are variations between countries in case definitions and completeness of reporting, topics that are discussed in further detail in the Discussion section of this paper.

**Table 1:** Data sources, data availability and definitions—COVID-19 cases.

Country or sub-national region	Institution holding data on COVID-19 cases	Definition of a confirmed COVID-19 case	Available time period (SEP 2022)	Time unit	Data by sub-national regions
Albania	National Institute of Public Health of Albania (Ministry of Health and Social Protection)	WHO case definition^1^	03/2020–06/ 2022	Daily	Yes
Federation of Bosnia & Herzegovina^2^	Institute of Public Health of Federation Bosna and Herzegovina	WHO case definition^1^	03/2020–12/ 2021	Daily	No
Republic of Srpska^2^	Public Health Institute of the Republic of Srpska	SARS-CoV-2 PCR Positive	03/2020–12/2021	Daily	No
Kosovo^3^	National Institute of Public Health of Kosovo	WHO case definition^1^	03/2020–03/2022	Daily	Yes
Montenegro	Institute of Public Health of Montenegro	WHO case definition^1^	03/2020–05/2022	Daily	Yes
Serbia	Institute of Public Health of Serbia	WHO case definition^1^	03/2020–07/2022	Daily	No
Türkiye	Ministry of Health	WHO case definition^1^	03/2020–05/2022	Daily	Yes
Azerbaijan	Ministry of Health İnformation and Statistics Center	SARS-CoV-2 PCR Positive	03/2020–06/2022	Daily	Yes
Georgia	Ministry of Health (Health Support Agency)	SARS-CoV-2 PCR Positive	03/2020-03/2022	Daily	Yes
Kazakhstan	Ministry of Health	WHO case definition^1^	03/2020–12/ 2021	Daily	No
Kyrgyzstan	Ministry of Health (e-Health)	SARS-CoV-2 PCR Positive	03/2020–12/ 2021	Monthly	No
Mongolia	Health Development Center, Ministry of Health of Mongolia	SARS-CoV-2 PCR Positive	03/2020–12/ 2021	Daily	Yes
Ukraine	Public Health Center of the Ministry of Health of Ukraine	WHO case definition^1^	03/2020–12/ 2021	Daily	Yes
Uzbekistan	Ministry of Health	SARS-CoV-2 PCR Positive	03/2020–12/ 2021	Daily	No

^1^ WHO case definition: SARS-CoV-2 PCR Positive OR meets clinical and/or epidemiological criteria with a positive SARS-CoV-2 antigen rapid diagnostic test, see https://apps.who.int/iris/rest/bitstreams/1453227/retrieve

^2^ Autonomous entity of Bosnia and Herzegovina

^3^ This designation is without prejudice to positions on status, and is in line with UNSCR 1244 and the ICJ Opinion on the Kosovo Declaration of Independence.

**Table 2:** Data sources, data availability and definitions—COVID-19 deaths.

Country or sub- national region	Data source	Institution holding data on COVID-19 deaths	Definition of a COVID-19 death	Available time period (SEP 2022)	Time unit	Data by sub-national regions
Albania	National surveillance data	National Institute of Public Health of Albania (Ministry of Health and Social Protection)	WHO definition^1^	03/2020–06/2022	Daily	Yes
Federation of Bosnia & Herzegovina^2^	Civil Registration / Vital Statistics	Federal Office of Statistics (Federation of Bosnia and Herzegovina)	WHO definition^1^	03/2020–12/2021	Monthly	No
Republic of Srpska^2^	National surveillance data	Public Health Institute of the Republic of Srpska	WHO definition^1^	03/2020–12/2021	Daily	No
Kosovo^3^	National surveillance data	National Institute of Public Health of Kosovo	WHO definition^1^	03/2020–03/2022	Daily	Yes
Montenegro	National surveillance data	Institute of Public Health of Montenegro	WHO definition^1^	03/2020—May 2022	Daily	Yes
Serbia	Civil Registration / Vital Statistics	Statistical Office of the Republic of Serbia	WHO definition^1^	03/2020–12/2021	Monthly	No
Türkiye	National surveillance data	Ministry of Health	WHO definition^1^	03/2020 - 31May 2022	Daily	Yes
Azerbaijan	National surveillance data	Ministry of Health information and Statistics Center	WHO definition^1^	03/2020–06/2022	Daily	Yes
Georgia	Civil Registration / Vital Statistics	Geostat/NCDC Georgia	WHO definition^1^	03/2020–12/2021	Daily	No
Kazakhstan	Ministry of Health data	Ministry of Health	WHO definition^1^	03/2020–12/2021	Monthly	No
Kyrgyzstan	Civil Registration / Vital Statistics	National Statistic Committee	WHO definition^1^	03/2020–12/2021	Monthly	No
Mongolia	National surveillance data	Health Development Center, Ministry of Health of Mongolia	WHO definition^1^	03/2020–12/2021	Daily	Yes
Ukraine	National surveillance data	Public Health Center of the Ministry of Health of Ukraine	WHO definition^1^	03/2020–12/2021	Daily	Yes
Uzbekistan	National surveillance data	Ministry of Health	WHO definition^1^	03/2020–12/2021	Daily	No

^1^ WHO definition of COVID-19 death: probable or confirmed SARS-CoV-2 infection with a clinically compatible illness and no alternative cause of death: https://www.who.int/publications/m/item/international-guidelines-for-certification-and-classification-(coding)-of-covid-19-as-cause-of-death

^2^ Autonomous entity of Bosnia and Herzegovina

^3^ This designation is without prejudice to positions on status, and is in line with UNSCR 1244 and the ICJ Opinion on the Kosovo Declaration of Independence.

To achieve its goals, the present study compiles these data sources, and brings them together to enable burden of disease estimations to be made. The calculations require COVID-19 specific statistics (i.e. data from disease monitoring and surveillance systems) as well as other estimated or registered population-level data inputs (such as cause of death statistics, life expectancy and population size). Prior to the data provision and analyses all information is aggregated in a way that no individual medical data are collected and processed. To achieve the study’s goal the underlying data is only provided disaggregated by age, sex, and over time. In using data that were initially collected by third parties for purposes such as surveillance and official statistical reporting, the study implemented in BoCO-19 can be described as a secondary data analysis, using existing information to achieve new research objectives.

#### Data collection

For all partners, aggregated data are collected for a minimum period of 12 months, from March 2020 until December 2021. However, some partners have access to data for a longer period of time (i.e. beyond February 2021). Data on population size, and where available—national life expectancies, are also collected from the relevant national statistical offices. The BoCO-19 project coordination developed data collection templates to instruct partners involved in gathering data in a logical and unambiguous way (S1 File). Partners use the template as a research instrument for collecting data on COVID-19 cases and deaths disaggregated by sex, and by either five- or ten-year age-groups. Partners are additionally asked to collect data disaggregated by month and if possible by sub-national level. The minimum (by age, sex, year) was set in order to be able to estimate the age and gender specific disease burden of COVID-19, as well as trends over time. Where partners cannot access the desired minimum level of disaggregation (i.e. at least by age, sex and time), assumptions will be made in order to fill the data gaps, based on publicly available information and scientific evidence. For additional excess mortality analyses a second data collection template was created (S2 File), to gather country specific all-cause mortality data (see section on Data Analysis below).

#### Data management and data protection

All data collection and processing are carried out by the individual partner institutions and is subject to the respective national data protection regulations. For the planned analyses no individual level data are collected as only information disaggregated by sex, age-group and region (where available) is required. For the purpose of cross-country comparisons and publications only the calculated results (YLL, YLD, DALY) will be integrated into a common results data base. Therefore, partner institutions and data holders retain ownership and control over their data at all times.

### Data analysis: Calculation of burden of disease indicators

The burden of COVID-19 will be calculated for each of the participating countries and sub-national regions over a minimum period of 12 months (March 2020 to February 2021), and comparisons will be made across countries and over time.

**Fig 3:**
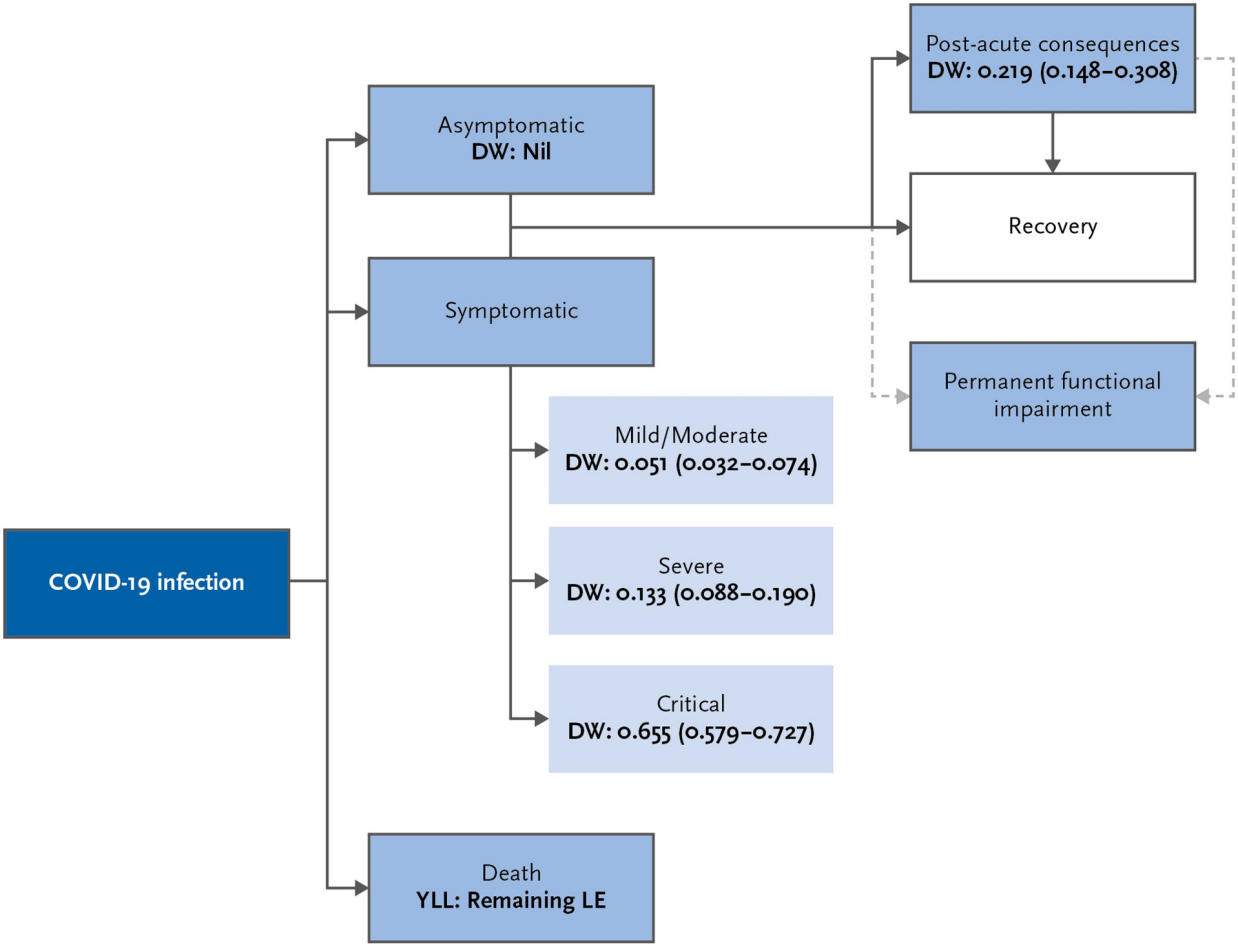
Basic disease model for calculating of the burden COVID-19, European Burden of Disease Network [29]. DW = Disability Weight; LE = Life Expectancy.

In late 2020, the EU COST Action CA18218 (the European Burden of Disease Network) proposed a consensus model for assessing the burden of disease COVID-19 (hereafter referred to as the Burden-EU model) (Fig 3). This disease model provides recommendations concerning data inputs, methodological decisions, and adaptations which can be made to the model. For the study implemented in BoCO-19, the Burden-EU model will be used as the starting point for calculating burden of disease indicators, with some adaptations relating primarily to the calculation of YLD that are addressed in more detail below.

#### Assessing health losses due to mortality: Calculation of YLL

YLL will be calculated as described in the Burden-EU model, which applies standard GBD methodology, by comparing a person’s age at the time of death with their residual life expectancy. All deceased individuals are assigned some years of residual life expectancy, regardless of their age at death (i.e. even an individual who dies aged 100 or older is assigned some remaining years of life expectancy). The following formula is used:

$$YLL=\sum_{i=0}^{n}\;d_{i,g}*l_{i}$$ (1)

where *i* is age-group in years, *d*_*i*,*g*_ the number of deaths in age-group *i* among persons with sex *g*, and *l*_*i*_ the residual life expectancy in age-group *i*.

Various life expectancy tables are available to choose from when doing YLL calculations, including national life expectancy estimates, as well as global ‘aspirational’ life tables compiled by international organisations such as the WHO or the Institute for Health Metrics and Evaluation (IHME). For example, the GBD Study 2019 created a global aspirational life expectancy table, which is based on the lowest observed age-specific mortality rates in all countries with populations over 5 million in 2016 [30]. Due to their better suitability for international comparison, for the proposed study these life expectancy tables will be used for calculation of YLL.

#### Scenario analysis: Excess mortality to assess potential underestimation of YLL

It is probable that COVID-19 deaths are—to different degrees in different countries or regions—under- or in some countries even overestimated [31–35]. Underestimation may be due to limited testing capacities or misclassifications of the cause of death, resulting in undetected COVID-19 deaths. Additionally, errors or insufficiencies in reporting infrastructure and different case definitions may mean that detected deaths are not equally or adequately covered by existing surveillance systems. In this respect, the total number of deaths in a country (all-cause mortality) and its variation over time may be of use to additionally estimate the impact of COVID-19 on population health. Various methods are available for performing such excess mortality calculations for the COVID-19 pandemic [16, 17, 36, 37]. In general, the excess is measured by comparing the number of all-cause deaths in pandemic years to all-cause deaths that would have been expected for that period given a baseline period, usually the three to five years preceding the start of the pandemic. Besides an expected increase in COVID-19 and other causes of death resulting in more deaths, a decrease in some causes of death is also possible. This has, for example, been observed in Germany for road traffic accidents [38]. Excess mortality estimates–based on all-cause mortality data–are therefore only used in a complementary manner in this analysis.

Existing excess mortality calculations for the countries included in BoCO-19 do not provide combined disaggregation by age, sex and over time, meaning the measurements are rather rough and undynamic [16, 39]. Different methodological choices have to be made when estimating excess mortality, and general all-cause mortality trends over time should be taken into account [37]—an aspect which will be incorporated into the calculations as part of the study that is implemented in BoCO-19. A straightforward approach will be used to ensure broad coverage of all countries, and the (possible) number of excess deaths and resulting YLL will be compared with the YLL based on officially reported COVID-19-deaths—over time and disaggregated by age and sex. This allows one to assess and discuss the possible underestimation of COVID-19-YLL in a population. By extending the database for the partner countries and regions, and exploring trends at a granular level, these estimations contribute to a better understanding of the overall population health loss resulting from the COVID-19 pandemic.

#### Assessing the health loss due to morbidity: Calculation of YLD

According to standard GBD methodology, YLD are calculated by summing up the severity grade specific products of the number of cases with the corresponding disability weight and duration of illness according to the following formula:

$$YLD=\;\overset{10}{\underset{i=1}{\sum }}\;\overset{2}{\underset{g=1}{\sum }}\;\overset{5}{\underset{j=1}{\sum }}\;\delta_{j}*I_{i,g,j}*DW_{j}$$ (2)

where *i* is the age-group, *g* the sex, *j* the grade of severity, *I*_*i*,*g*,*j*_ the number of reported cases and *DW*_*j*_ the specific disability weight for grade of severity *j*. An additional multiplicative factor is used to reflect the duration of illness: $\delta_{j}=\frac{duration_{j}}{365.25}$ with durations measured in days with 365,25 reflecting the average days per year.

The Burden-EU disease model defines four different severity grades of COVID-19 illness (mild/moderate, severe, critical and post-acute consequences—the last category including long COVID) [28, 29], with corresponding disability weights which are based on GBD 2019 values for infectious diseases of the lower respiratory tract and the European Disability Weights Study (Fig 3). If the data allow, mild cases (e.g. patients with mild cold symptoms, but no fever) can be separated from moderate cases (e.g. patients with fever or pneumonia in outpatient treatment) and assigned their own, significantly lower disability weight (0.006) [20]. A special aspect in measuring the burden of COVID-19 are the post-acute consequences of the disease. Since the start of the pandemic, there has been continually emerging evidence on this topic, including which sequelae occur and how frequently [40]. In addition to a prolonged duration of typical symptoms (long COVID), other possible post-acute consequences include permanent functional impairment—a severity grade which is referred to in the Burden-EU disease model, but was not used in many burden of COVID-19 studies conducted to date, due to the newly emerging state of the scientific evidence [28]. After review of the current literature, the burden of long COVID will be likely considered adopting an evidence-based approach that has been recently suggested by IHME for the GBD study [40]. In differentiating three broad symptomatic areas (respiratory symptoms, cognitive symptoms, fatigue syndrome), and by proposing parameters regarding incidence, disability weights and durations for these sequelae, this approach goes far beyond the rather rough implementations of the Burden-EU model (e.g. [23, 26, 27]).

In many existing national burden of disease studies, the distribution of the diseased population across the various health states is measured using country-specific data, though due to data limitations this is not always possible, and severity distributions must sometimes be applied in a uniform way across countries or population groups [41]. Within BoCO-19, data to empirically measure country-specific COVID-19 severity distributions and durations are only available for a few countries. Therefore, the possibility and suitability of applying a uniform severity distribution—for example using data from one of the partner countries or based on German data—will be explored. Measuring the ‘true’ severity distribution of cases is challenging and depends on many factors such as testing rates, the prevalence of other chronic illnesses in the population, as well as healthcare system infrastructures and capacities, so that the strengths and weaknesses of applying any single uniform severity distribution must be carefully considered.

According to data availability, a distinction between mild and moderate cases—and thus the attribution of different disability weights—may be possible. Furthermore, since the underlying data are available for the entire duration of the pandemic disaggregated by time, different distributions could be approximated according to the prevailing virus variant (Alpha, Delta, Omicron) and the vaccination coverage. To apply this information to different countries and regions, the temporal delimitation of the individual waves would have to be provided by the partner institutions on the basis of the nationally available evidence. Whether such a disaggregation of severity distributions by different pandemic periods is already indicated for the reference period of the study will be finally decided after a close inspection of the severity trends.

Severity specific durations, except for post-acute consequences, were calculated for a German analysis for 2020 (14 days for asymptomatic, mild and moderate cases, 21 days for severe cases and 32 days for critical cases) [20]. These durations have been empirically confirmed in an Australian analysis and are therefore considered to remain valid and applicable [25].

Finally, as part of the YLD calculations, the feasibility of correcting for under-reporting of infected cases will be examined. Various methods for this are available [42], making use for example of national estimates of rates or under-reporting from sero-prevalence studies, and some approaches have already been applied in calculating the disease burden of COVID-19 [21, 26, 27]. The extent to which relevant national data are available, and to which existing approaches can be applied to the present analyses, will be explored.

#### Assessing the combined health losses due to morbidity and mortality: Calculation of DALY

DALY estimates for each of the partner countries result from summing up YLL and YLD.

### Ethical considerations

At the Robert Koch Institute as a government agency, the legal department assumes the role of an Institutional Review Board in the ethical review of non-interventional studies that do not themselves collect medical data on individuals. In BoCO-19 only aggregated data is used that has been collected by governmental agencies as part of a mandatory reporting on notifiable diseases. Hence, BoCO-19 uses existing information on COVID-19 cases and deaths in the population that is disaggregated only by age, sex and time prior to the data provision and analysis. Such data on infectious diseases are used to describe and assess the epidemiological situation and are forwarded to international bodies such as the WHO in order to analyse population health on an international level. No additional individual data on humans is collected and interventions are neither feasible nor planned. Hence, the data used in BoCO-19 are official aggregated case counts and not individual medical information. The legal department of the Robert Koch Institute has therefore stated that an ethical vote is not required for the data usage in question (Reference No. 1.09.04/0011#0259).

## Discussion

Based on a harmonized methodology, the present protocol describes the implementation of a study aimed at assessing and comparing the burden of COVID-19 across multiple countries and regions. Further supporting activities that are implemented within BoCO-19, like capacity building and training workshops for the implementation of burden of disease methods, are described. In addition to fostering international cooperation and the creation of policy relevant results, there are also some challenges in implementing the proposed methodology and interpreting the results, which are discussed in more detail below.

### Challenges and limitations of the study

A number of challenges present themselves in terms of interpreting and comparing the results generated by the study that is implemented in BoCO-19. This includes that there will be differences between countries in terms of how COVID-19 cases and deaths are defined and counted. With regard to the definition of COVID-19 cases, representing data that feed into YLD calculations, half of the partners within the BoCO-19 consortium count only those with a positive PCR test as confirmed cases, while in the other seven countries cases with a positive rapid diagnostic test, but which also fill other epidemiological or clinical criteria, are also included. In addition, official death counts reported by national authorities may reflect limitations in access to healthcare and in counting deaths in out-of-hospital settings. As a result, the degree to which case counts represent under- or overestimates will vary between countries, depending on the sensitivity of the case definitions used, and also on differences in healthcare systems and in testing capacities and strategies [43]. With regard to COVID-19 deaths, the WHO recommends that these should include cases with a probable or confirmed SARS-CoV-2 infection, where there was a clinically compatible illness and no alternative cause of death [44]. However, the precise implementation of this recommendation may vary, and how a COVID-19 death is defined or assigned may depend on the data source, i.e. whether deaths data are acquired from national surveillance systems (the case for 10 BoCO-19 partners), or civil registration and vital statistics systems (the case for four BoCO-19 partners)–the latter system often following protocols which stipulate that only one main underlying cause can be assigned per death. Conversely in some surveillance systems, deaths among patients who had COVID-19, but for whom it was not the primary underlying cause of death, are still counted. A potentially larger issue relates to under-reporting of deaths, i.e. true COVID-19 deaths which are not reported into any system at all. However, excess mortality calculations will provide some measure of the extent of this potential underestimation, and will be important to consider alongside the YLL results, bearing in mind that excess mortality cannot be attributed to the pandemic alone and that changes in trends in other causes of death may also take place. Such may be diminishing road traffic accidents during lock-downs or, conversely potentially increased mortality due to factors such as delayed treatment of non-communicable diseases.

In general, different definitions of COVID-19 cases and deaths as well as their implementation in practice will be dealt with in subsequent publications in such a way that the most important differences will be made as transparent as possible and limitations in the interpretation of results will be pointed out.

In addition, two central methodological decisions in the calculation of YLL and YLD also lead to basic assumptions that entail various advantages and disadvantages. An advantage of employing a life expectancy table such as GBD 2019 when calculating YLL is that it enables international comparisons to be made, as YLL for all countries are calculated using a single standard of the highest empirically achievable life expectancy at the age of death at the global level. Disadvantages of such an approach include that the YLL calculated no longer represent the burden based on the life expectancy that is empirically realistic for a single country and thus tend to overestimate the YLL for a specific country. Some partners will additionally perform calculations using national life expectancy tables, where available, in order to provide a measure of YLL that is closer to the mortality situation in their country. Results based on national life expectancy tables may be potentially better suited to communicate core findings of the study to stakeholders and the public within individual countries.

For the calculation of YLD, the use of uniform severity distributions and durations of illness across all countries may be a pragmatic solution, because in many countries data or capacities to calculate national severity distributions or durations are unavailable. A disadvantage of such an approach is that it may apply a severity distribution in each country which deviates to some extent from the ‘true’ distribution of cases in that population [41]. Furthermore, differences in the performance of individual health systems are masked by the application of a uniform severity distribution. However, making this visible is not the aim of burden of disease analyses. Severity distributions, even if key figures such as hospitalization are used, reflect basic epidemiological assumptions. Therefore, it may even be seen as an advantage of this approach that the effect of differences between individual health systems—in terms of the available infrastructure as well as differences in how healthcare-related decisions are made—is minimized. Notwithstanding the advantages and disadvantages of the approach, it is important to emphasise that YLD usually account for a much smaller proportion of the overall burden due to COVID-19 when compared to YLL [20, 21, 27]. Provided that this ratio is not clearly reversed by new virus variants with a much higher impact of morbidity, e.g. due to more frequent and severe post-acute consequences, it can be assumed that the choice of severity distribution is likely to have a limited impact on the overall burden of disease caused by COVID-19.

### Dissemination of results

Findings from the study that is implemented in BoCO-19 will be disseminated in a variety of ways subject to the consent of the individual partners to participate in the respective forms of dissemination. Further to this study protocol, providing that data quality and comparability allow, additional scientific papers will strive to present the YLL, YLD, and DALY results from partner countries, enabling comparisons across countries. The results will be shared widely with relevant stakeholders in the partner countries, as well as through the European Burden of Disease Network. Additionally, partners are supported to discuss individual country-specific analyses and how the results from the project might contribute to improved data quality and reporting in their regions. A manual with guidance relating to the integration of burden of disease indicators into disease monitoring and surveillance systems is planned that will highlight the comparative benefit of estimating burden of disease indicators, an overview of data requirements, guidance to potentially improve data quality by defining for instance common case definitions, and a description of the required personnel resources.

## Conclusion

BoCO-19 is a collaborative platform, bringing together public health experts from 14 different countries and sub-national regions across Southern and Eastern Europe and Central Asia, in order to develop, harmonize and apply methodologies for the calculation of the burden of COVID-19. As the need for reliable data and public health indicators has become abundantly clear, particularly in the wake of the COVID-19 pandemic, the secondary benefits of such a collaboration, in terms of its ability to strengthen capacities for the calculation and interpretation of burden of disease indicators, are exceedingly high. Apart from the better use of available data, this includes jointly developing strategies to detect and deal with data quality issues (for example, under- or overestimation of cases). Other aspects considered as part of BoCO-19 include improving the presentation, visualization, dissemination, and communication of results as well as mutual learning to better cope with future health emergencies. Burden of disease indicators can contribute to timely surveillance of dynamic outbreaks and help to monitor the impact of policy measures. The pandemic revealed the limitations of many existing surveillance and reporting systems at a time when they were most needed. Analyses over time, as in BoCO-19, illustrate the dynamics of pandemics and therefore the benefits of making data available in a timely manner.

## Supporting information

S1 File Data collection template COVID-19 cases and deaths. (XLSX)

S2 File Data collection template all cause deaths. Where appropriate for a study protocol, the Guidelines for Accurate and Transparent Health Estimates Reporting (GATHER, http://gather-statement.org/) have been considered in the preparation of this article [45].
(XLSX)

S3 File GATHER checklist. (PDF)
